# Increased 90-kDa ribosomal S6 kinase (Rsk) activity is protective against mutant huntingtin toxicity

**DOI:** 10.1186/1750-1326-6-74

**Published:** 2011-10-31

**Authors:** Xavier Xifró, Marta Anglada-Huguet, Laura Rué, Ana Saavedra, Esther Pérez-Navarro, Jordi Alberch

**Affiliations:** 1 Departament de Biologia Cel·lular, Immunologia i Neurociències, Facultat de Medicina, Universitat de Barcelona, Spain; 2 Institut d'Investigacions Biomèdiques August Pi i Sunyer (IDIBAPS), Barcelona, Spain; 3 Centro de Investigación Biomédica en Red sobre Enfermedades Neurodegenerativas (CIBERNED), Spain; 4 Departament de Ciències Mèdiques, Facultat de Medicina, Universitat de Girona, Spain

**Keywords:** cell death, ERK, Huntington's disease, knock-in mouse, neuroprotection, PDK1, R6/1 mouse, striatum

## Abstract

**Background:**

The 90-kDa ribosomal S6 kinase (Rsk) family is involved in cell survival. Rsk activation is regulated by sequential phosphorylations controlled by extracellular signal-regulated kinase (ERK) 1/2 and 3-phosphoinositide-dependent protein kinase 1 (PDK1). Altered ERK1/2 and PDK1 phosphorylation have been described in Huntington's disease (HD), characterized by the expression of mutant huntingtin (mhtt) and striatal degeneration. However, the role of Rsk in this neurodegenerative disease remains unknown. Here, we analyzed the protein levels, activity and role of Rsk in *in vivo *and *in vitro *HD models.

**Results:**

We observed increased protein levels of Rsk1 and Rsk2 in the striatum of Hdh^Q111/Q111 ^and R6/1 mice, STHdh^Q111/Q111 ^cells and striatal cells transfected with full-length mhtt. Analysis of the phosphorylation of Rsk in Hdh mice and STHdh cells showed reduced levels of phospho Ser-380 (dependent on ERK1/2), whereas phosphorylation at Ser-221 (dependent on PDK1) was increased. Moreover, we found that elevated Rsk activity in STHdh^Q111/Q111 ^cells was mainly due to PDK1 activity, as assessed by transfection with Rsk mutant constructs. The increase of Rsk in STHdh^Q111/Q111 ^cells occurred in the cytosol and in the nucleus, which results in enhanced phosphorylation of both cytosolic and nuclear Rsk targets. Finally, pharmacological inhibition of Rsk, knock-down and overexpression experiments indicated that Rsk activity exerts a protective effect against mhtt-induced cell death in STHdh^Q7/Q7 ^cells transfected with mhtt.

**Conclusion:**

The increase of Rsk levels and activity would act as a compensatory mechanism with capacity to prevent mhtt-mediated cell death. We propose Rsk as a good target for neuroprotective therapies in HD.

## Background

The 90-kDa ribosomal S6 kinase (Rsk) is a family constituted by four isoforms (Rsk1-4) of serine/threonine kinases broadly expressed in the brain that regulate important cellular functions, including cell survival [1]. Rsk is activated by extracellular signal-regulated protein kinase (ERK) 1/2 [2] and 3-phosphoinositide-dependent protein kinase 1 (PDK1) [3] by sequential phosphorylations in the C-terminal kinase domain (CTKD) and N-terminal kinase domain (NTKD) [1,4], respectively. Briefly, sequential phosphorylations are initiated by ERK1/2 at Thr-573/574 of CTKD leading to the auto-phosphorylation of Rsk at Ser-380. This phosphorylation allows the dockage of PDK1 to the hydrophobic motif and enables PDK1-dependent phosphorylation in the NTKD of Rsk at Ser-221, resulting in its maximal activation [1,4]. When activated, Rsk promotes the phosphorylation of many cytosolic and nuclear targets. In the cytosol, Rsk induces the inactivation of certain pro-apoptotic proteins, such as Bad [5], glycogen synthase kinase 3β (GSK-3β) [6] or death-associated protein kinase (DAPK) [7], whereas in the nucleus it activates transcription factors involved in the synthesis of anti-apoptotic proteins, namely cAMP response element binding protein (CREB) [8], serum response factor (SRF) [9], and IκBα [10,11]. Although the function and the mechanism of Rsk activation have been well studied in non-neural cells, in neurons there are few studies about Rsk, and they associate its activity with the anti-apoptotic effect of trophic factors [12-14]. However, no data exists about the possible role of Rsk in neurodegenerative diseases.

Huntington's disease (HD) is a neurodegenerative disorder caused by a dominantly heritable expansion of a trinucleotide CAG repeat in the *huntingtin *(htt) gene [15], and characterized by the preferential neurodegeneration of striatal medium-sized spiny neurons [16]. Although the brain areas affected by the disease are well established, the mechanisms by which neural dysfunction and neurodegeneration occurs are not well defined yet. Interestingly, previous data from a HD cellular model show a de-regulation of both kinases that control Rsk activity. Knock-in striatal cells expressing full-length mutant huntingtin (mhtt) (STHdh^Q111/Q111^) show increased levels of active PDK1 [17] and reduced levels of ERK1/2 activity [18] compared with striatal cells expressing wild-type htt (STHdh^Q7/Q7^). Moreover, stimulation of these kinases and their pathways has been proposed as good therapeutic approaches for HD [19-21]. These results suggest a de-regulation of Rsk activity in HD models and that modulation of its activity could be a good therapeutic strategy. Therefore, here we studied whether the protein levels and activity of Rsk1 and Rsk2, the two isoforms with higher expression levels [1], are modified in the presence of mhtt. To this end, we analyzed striatal protein levels and activity of Rsk in knock-in mhtt mouse and cellular models. In addition, we studied the contribution of ERK1/2 and PDK1 to the activation of Rsk in the presence of mhtt. Finally, we evaluated the potential protective role of Rsk against mhtt toxicity.

## Results

### Rsk1 and Rsk2 protein levels are increased in knock-in and R6/1 models of HD

**Figure 1:**
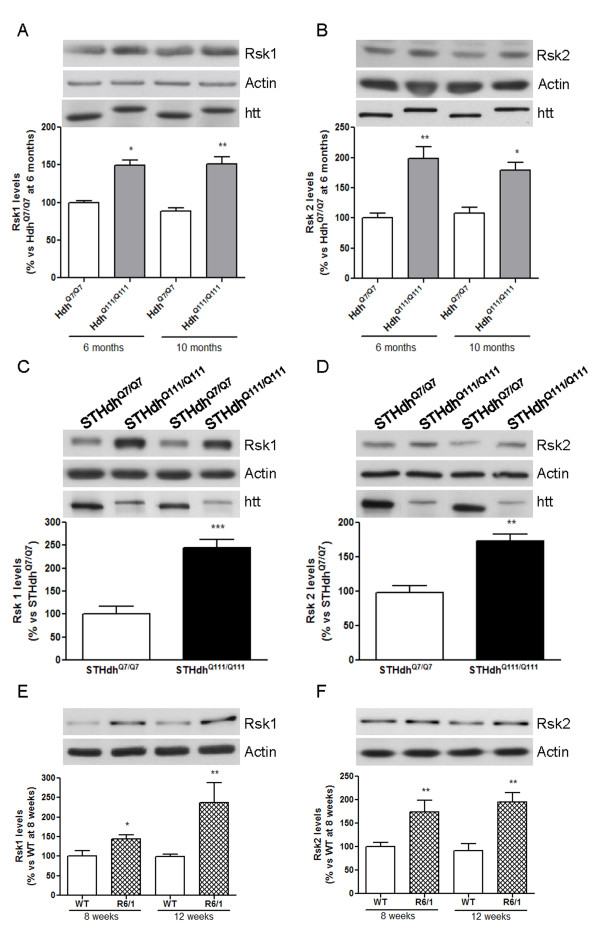
**Rsk levels are elevated in HD mouse and cellular models**. Rsk1 (A, C and E) and Rsk2 (B, D and F) protein levels were analyzed by western blot of protein extracts obtained from the striatum of 6- and 10-month old wild-type (Hdh^Q7/Q7^) and knock-in (Hdh^Q111/Q111^) mice (A and B), from wild-type (STHdh^Q7/Q7^) and mutant (STHdh^Q111/Q111^) htt knock-in striatal cells (C and D), and from the striatum of 8- and 12-week old WT and R6/1 mice (E and F). Htt protein levels were also analyzed by western blot in knock-in models (A-D). Results (mean ± SEM; n = 4-6) represent the ratio between Rsk and actin levels obtained by densitometric analysis of western blot data, and are expressed as a percentage of Hdh^Q7/Q7 ^levels at 6 months (A and B), as a percentage of protein levels in STHdh^Q7/Q7 ^cells (C and D), or as a percentage of protein levels in WT mice at 8 weeks (E and F). Data were analyzed by two-way ANOVA followed by Bonferroni's *post hoc *test (A, B, E and F) or by Student's t-test (C and D). *p < 0.05 and **p < 0.01 as compared with Hdh^Q7/Q7 ^mice (A and B), **p < 0.01 and ***p < 0.001 as compared with STHdh^Q7/Q7 ^cells (C and D), and *p < 0.05 and **p < 0.01 as compared with WT mice (E and F). Representative immunoblots are presented.

First of all, we analyzed by western blot whether the protein levels of the two major Rsk isoforms, Rsk1 and Rsk2, were altered in the striatum of 6- and 10-month old wild-type (Hdh^Q7/Q7^) and mutant (Hdh^Q111/Q111^) knock-in mice. We observed elevated Rsk1 and Rsk2 protein levels in the striatum of Hdh^Q111/Q111 ^mice compared to Hdh^Q7/Q7 ^mice at both ages (Figure 1A and 1B). In addition, the levels of these proteins were also augmented in striatal cells expressing full-length mhtt (STHdh^Q111/Q111^) when compared to those in wild-type cells (STHdh^Q7/Q7^; Figure 1C and 1D). These changes in Rsk1 and Rsk2 protein levels were not dependent on mhtt protein levels since we observed increased levels in Hdh mouse striatum expressing similar levels of mhtt (Figure 1A and 1B; Hdh^Q7/Q7 ^mice: 100 ± 9.8%; Hdh^Q111/Q111 ^mice: 89 ± 10.3%; Student's *t*-test: p = 0.4164) and in STHdh^Q111/Q111 ^cells, which express lower levels of mhtt compared with STHdh^Q7/Q7 ^(Figure 1C and 1D; STHdh^Q7/Q7 ^cells: 100 ± 11.6%; STHdh^Q111/Q111 ^cells: 36 ± 6.9%; Student's *t*-test: p < 0.001).

To know whether increased Rsk1 and Rsk2 protein levels also occur in exon-1 mhtt mice we analyzed by western blot these proteins in the striatum of R6/1 mice at 8- and 12-week of age, when they do not show motor symptoms [22]. Similar to that observed in the striatum of knock-in mice, R6/1 mouse striatum displayed higher Rsk1 and Rsk2 levels compared to wild-type (WT) mice at both ages (Figure 1E and 1F). Altogether, these results indicate that an increase of Rsk1 and Rsk2 protein levels is an event that occurs in full-length and exon-1 models of HD at presymptomatic stages. In addition, changes in Rsk1 and Rsk2 are not dependent on mhtt levels since we observed a similar response in striatal cells expressing low (STHdh^Q111/Q111 ^cells), normal (knock-in mice striatum) or very high (R6/1 mouse striatum) levels of mhtt.

### Overexpression of full-length mhtt increases Rsk protein levels

**Figure 2:**
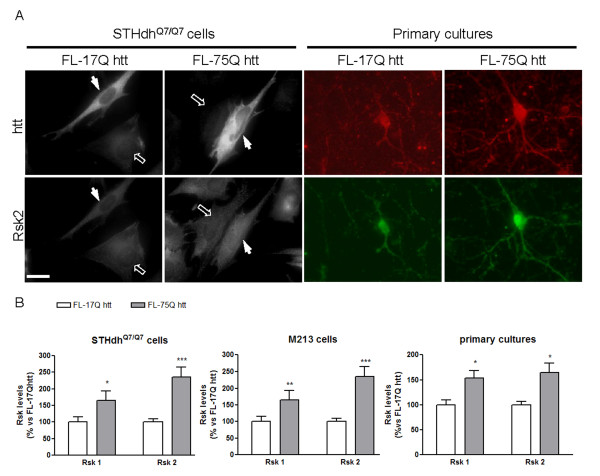
**Overexpression of full-length mhtt in striatal cells increases Rsk levels**. (A) Representative photomicrographs of htt and Rsk2 immunoreactivity in STHdh^Q7/Q7 ^cells and striatal primary cultures after transfection (48 hours and 24 hours, respectively) with full-length wild-type (FL-17Q htt) or mutant (FL-75Q htt) htt. Closed arrows indicate transfected cells (cells expressing higher levels of htt) and open arrows indicate non-transfected cells. Scale bar: 16 μm. (B) Graphs showing Rsk1 and Rsk2 protein levels measured in STHdh^Q7/Q7 ^cells, M213 cells and striatal primary cultures after transfection (48 hours for STHdh^Q7/Q7 ^and M213 cells, and 24 hours for striatal primary cultures) with full-length wild-type (FL-17Q htt) or mutant (FL-75Q htt) htt. Data were expressed in arbitrary units and are the mean ± SEM of three independent experiments performed in triplicate. Data were analyzed by Student's *t*-test. *p < 0.05; **p < 0.01 and ***p < 0.001 as compared with FL-17Q htt-transfected cells.

In order to confirm that increased levels of Rsk were dependent on mhtt expression, we looked at the protein levels of Rsk1 and Rsk2 in STHdh^Q7/Q7 ^cells, M213 cells and striatal primary neurons transfected with a plasmid expressing full-length wild-type (FL-17Q htt) or mutant (FL-75Q htt) htt. The quantification of Rsk1 and Rsk2 levels was performed by confocal microscopy due to the low efficiency of transfection (15-20% approximately). In all cell types examined, transfection with FL-75Q htt increased Rsk1 and Rsk2 protein levels compared to those registered in cells expressing FL-17Q htt (Figure 2), indicating that the increase in Rsk1 and Rsk2 protein levels are due to the presence of mhtt.

### Rsk phosphorylation in HD knock-in models: ERK-dependent residues *versus *PDK1-dependent residues

**Figure 3:**
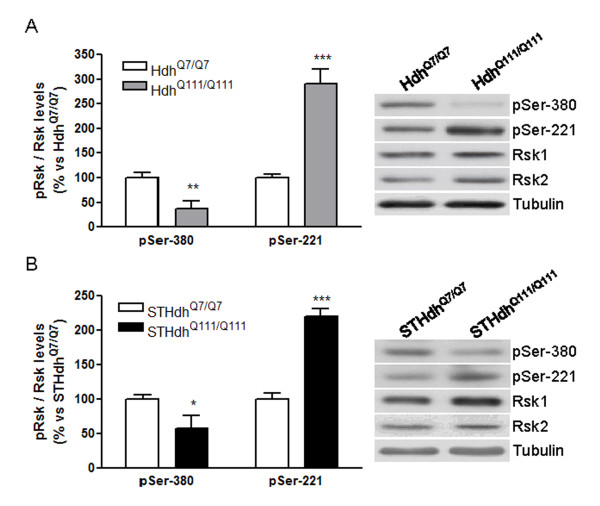
**Different regulation of phospho-Rsk residues in HD knock-in mice and STHdh^Q111/Q111 ^cells**. Lysates from the striatum of 10-month old wild-type (Hdh^Q7/Q7^) and knock-in (Hdh^Q111/Q111^) mice (A) or from wild-type (STHdh^Q7/Q7^) and mutant (STHdh^Q111/Q111^) htt knock-in striatal cells (B) were subjected to western blot to analyze phosphor-Rsk (Ser-380; Ser-221), Rsk1, Rsk2 and tubulin protein levels. Results (mean ± SEM; n = 4-6) represent the ratio between phospho-Rsk levels and Rsk1 plus Rsk2 levels obtained by densitometric analysis of western blot data, and are expressed as a percentage of Hdh^Q7/Q7 ^(A) or STHdh^Q7/Q7 ^(B) levels. Representative immunoblots are presented. All data were analyzed by Student's *t*-test. *p < 0.05, **p < 0.01 and ***p < 0.001 as compared with Hdh^Q7/Q7 ^mice (A) or STHdh^Q7/Q7 ^cells (B).

To study whether the phosphorylation levels of Rsk were altered by changes in total Rsk protein levels, we analyzed its phosphorylation at Ser-380 (dependent on ERK1/2) and at Ser-221 (dependent on PDK1), in the striatum of 10-month old Hdh^Q7/Q7 ^and Hdh^Q111/Q111 ^mice. We detected reduced levels of phospho-Rsk (Ser-380; reduction of 63 ± 13%; Figure 3A) and increased levels of phospho-Rsk (Ser-221; increase of 190 ± 19%; Figure 3A) in Hdh^Q111/Q111 ^respect to Hdh^Q7/Q7 ^mice. Similar results were obtained in STHdh^Q111/Q111 ^cells (Figure 3B). These results indicate that in the presence of mhtt the phosphorylation of Rsk at ERK- and PDK1-dependent residues is altered in an opposite way.

### STHdh^Q111/Q111 ^cells show increased Rsk activity that is mainly regulated by PDK1

**Figure 4:**
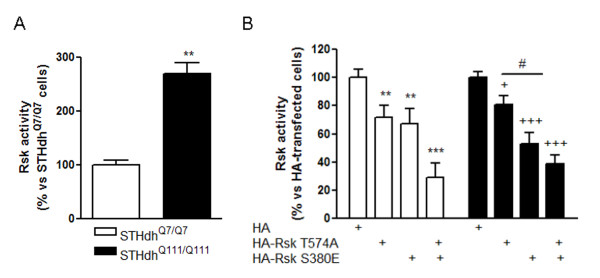
**Increased Rsk activity in STHdh^Q111/Q111 ^cells is mainly regulated by PDK1**. (A) Lysates from STHdh^Q7/Q7 ^and STHdh^Q111/Q111 ^cells were subjected to immunoprecipitation with anti-Rsk1 and anti-Rsk2 antibodies and the resulting immunocomplexes were used to determine Rsk activity as indicated in material and methods. Kinase activity was expressed as percentage of Rsk activity in STHdh^Q7/Q7 ^cells and data are the mean ± SEM of three independent experiments. Results were analyzed by Student's *t*-test. **p < 0.01 as compared with STHdh^Q7/Q7 ^cells. (B) STHdh^Q7/Q7 ^and STHdh^Q111/Q111 ^cells were transfected with the following constructs: HA-RskT574A (to study the role of ERK1/2 on Rsk activity) and HA-RskS380E (to analyze the role of PDK1 on Rsk activity). Twenty-four hours after transfection, both STHdh^Q7/Q7 ^and STHdh^Q111/Q111 ^cells were subjected to Rsk activity assay. Results are the mean ± SEM of three independent experiments and are expressed as percentage of control cells (cells transfected with HA alone). Data were analyzed by two-way ANOVA followed by Bonferroni's *post hoc *test. **p < 0.01 and ***p < 0.001 as compared with STHdh^Q7/Q7 ^control cells, ^+^p < 0.05 and^+++^p < 0.001 as compared with STHdh^Q111/Q111 ^control cells and^#^p < 0.05 as compared with STHdh^Q111/Q111 ^cells transfected with HA-Rsk T574A.

Our next goal was to know whether increased Rsk1 and Rsk2 protein and phosphorylation levels were associated with elevated Rsk activity. To this end, we analyzed Rsk activity in knock-in cells by using an *in vitro *activity assay. We observed that Rsk activity was higher in STHdh^Q111/Q111 ^than in STHdh^Q7/Q7 ^cells (270 ± 15%; Figure 4A). Moreover, overexpression of Rsk by transfection of HA-Rsk1 in STHdh^Q7/Q7 ^cells increased Rsk activity (STHdh^Q7/Q7 ^cells: 100 ± 9%; STHdh^Q7/Q7 ^+ HA-Rsk: 222 ± 13%; p < 0.0002; Student's *t*-test) indicating that one of the parameters that regulates Rsk activity is its protein levels.

To address the importance of phosphorylation by ERK1/2 and PDK1 on elevated Rsk activation in STHdh^Q111/Q111 ^cells, we measured Rsk activity in knock-in cells transfected with two mutant forms of Rsk: HA-RskT574A and HA-RskS380E, which cannot be phosphorylated by ERK1/2 and PDK1, respectively. Transfection with HA-RskT574A or HA-RskS380E similarly reduced Rsk activity in STHdh^Q7/Q7 ^cells (reduction of 28% and 33% respectively; Figure 4B). Interestingly, and supporting a main role for PDK1 in the increased Rsk activity observed in STHdh^Q111/Q111 ^cells, transfection with HA-RskS380E induced a stronger decrease of Rsk activity (47%; Figure 4B) compared with the transfection with HA-RskT574A (19%; Figure 4B). Note that co-transfection with both mutant forms reduced the activity of Rsk only by 30-40% probably because the efficiency of transfection was not maximal (Figure 4B).

### Rsk levels are increased in both cytosol and nucleus of STHdh^Q111/Q111 ^cells

**Figure 5:**
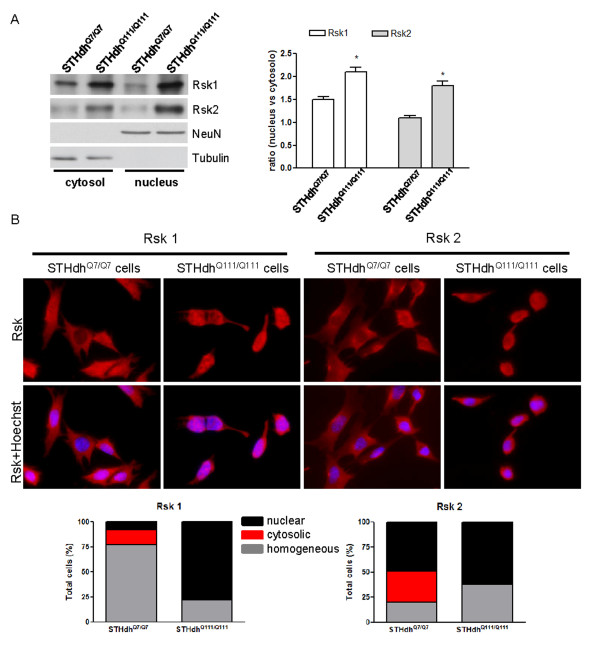
**Striatal cells expressing full-length mhtt show increased levels of Rsk in nuclear and cytosolic compartments**. (A) Rsk1, Rsk2, tubulin and NeuN were analyzed by western blot in cytosolic and nuclear fractions of STHdh^Q7/Q7 ^and STHdh^Q111/Q111 ^cells. Four independent experiments were performed in duplicate. Representative immunoblots are shown. We quantified the levels of Rsk1 and Rsk2 in both types of cells and analyzed the ratio between the nuclear and the cytosolic levels for each case. Data were analyzed by two-way ANOVA followed by Bonferroni's *post hoc *test. *p < 0.05 as compared with STHdh^Q7/Q7 ^cells. (B) Representative photomicrographs showing Rsk1 and Rsk2 immunoreactivity in STHdh^Q7/Q7 ^and STHdh^Q111/Q111 ^cells. The number of cells was quantified according to three different patterns of Rsk1 and Rsk2 localization: homogeneous localization (cytosolic and nuclear), exclusive cytosolic and exclusive nuclear localization. Nuclear localization was determined by co-staining with Hoechst 33258. Results are expressed as percentage of total cell number. Data was analyzed by chi-square (*χ*^2^). For Rsk1; χ square df: 102.5, 2 and p < 0.001. For Rsk2: χ square df: 38.11, 2 and p < 0.001.

Phosphorylated and activated Rsk can translocate from the cytosol to the nucleus. In these compartments, it regulates different targets [1]. Thus, we studied Rsk1 and Rsk2 levels in cytosolic and nuclear fractions of knock-in cells by western blot. When compared with control cells, STHdh^Q111/Q111 ^cells displayed enhanced levels of Rsk1 and Rsk2 in both compartments, with a more pronounced effect in the nucleus (Figure 5A). To confirm these data, we analyzed the localization of Rsk by immunocytochemistry. We detected three different patterns of expression: homogeneous expression, and exclusive cytosolic or nuclear localization (Figure 5B). Analysis of STHdh^Q7/Q7 ^cells revealed a predominant homogeneous distribution of Rsk1, whereas Rsk2 was mainly located in the nucleus. In STHdh^Q111/Q111 ^cells, Rsk1 changed its distribution as it was located only in the nucleus, while the nuclear expression of Rsk2 was even more evident than in STHdh^Q7/Q7 ^cells. Note that we did not observe exclusive cytosolic localization of either Rsk isoforms in STHdh^Q111/Q111 ^cells (Figure 5B). Altogether, these results indicate that although the increase of Rsk protein levels in STHdh^Q111/Q111 ^cells occurs in both compartments, this increase is more pronounced in the nucleus.

### Increased activity of Rsk in STHdh^Q111/Q111 ^cells enhances the phosphorylation of both cytosolic and nuclear targets

**Figure 6:**
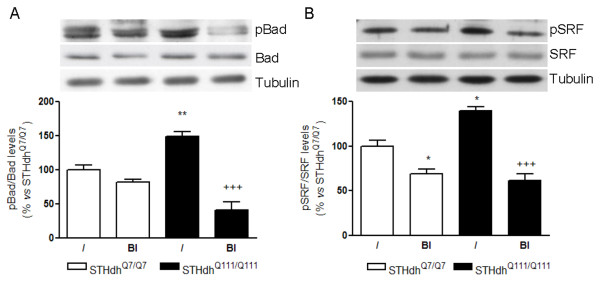
**Inhibition of Rsk induces a reduction of pBAD and pSRF levels in STHdh^Q111/Q111 ^cells**. Lysates from STHdh^Q7/Q7 ^and STHdh^Q111/Q111 ^cells treated with or without BI-D1870 (0.1 μM; BI) were subjected to western blot to analyze pBAD (Ser-112)/Bad (A) and pSRF (Ser-103)/SRF (B) and tubulin protein levels. Results are expressed as the ratio between phospho and total Bad (A) and SRF (B) levels. Data are the mean ± SEM of four independent experiments performed in duplicate. Representative immunoblots are presented. Results are expressed in percentage respect to STHdh^Q7/Q7 ^cells. Data were analyzed using two-way ANOVA followed by Bonferroni's *post hoc *test. *p < 0.05 and **p < 0.01 as compared with control STHdh^Q7/Q7 ^cells and^+++^p < 0.001 as compared with control STHdh^Q111/Q111 ^cells.

Rsk plays its protective role through the inactivation of cytosolic pro-apoptotic proteins and/or the activation of transcription factors that mediate the synthesis of anti-apoptotic proteins. Thus, we determined in knock-in cells expressing wild-type or mhtt the phospho-levels of two Rsk substrates, Bad at Ser-112 (cytosolic target), and SRF at Ser-103 (nuclear target). According with the elevated Rsk activity observed in STHdh^Q111/Q111 ^cells, we found increased levels of phospho-Bad (Figure 6A) and phospho-SRF (Figure 6B) respect to STHdh^Q7/Q7 ^cells. Then, to determine whether the increased phosphorylation of Bad and SRF was due to the action of Rsk, we treated knock-in cells with a pharmacological and specific inhibitor of Rsk, BI-D1870 (0.1 μM) [23]. The presence of BI-D1870 reduced the phosphorylation levels of both Bad and SRF in STHdh^Q111/Q111 ^cells (Figure 6A and 6B). In STHdh^Q7/Q7 ^cells, we did not observe changes in phospho-Bad levels in the presence of BI-D1870 (Figure 6A), whereas phospho-SRF levels were slightly decreased (Figure 6B). To corroborate that BI-D1870 efficiently inhibited Rsk, we tested the activity of Rsk in both cell lines after treatment with Rsk inhibitor. Addition of BI-D1870 (0.1 μM) completely inhibited Rsk activity in STHdh cells (STHdh^Q7/Q7 ^cells: 100 ± 14%; STHdh^Q7/Q7 ^+ BI: 7 ± 3%; STHdh^Q111/Q111 ^cells: 286 ± 22%; STHdh^Q111/Q111 ^+ BI: 13 ± 5%). These results show that increased Rsk activity in STHdh^Q111/Q111 ^cells results in augmented phosphorylation of both cytosolic and nuclear targets.

### Increased Rsk activity contributes to prevent mhtt-induced cell death

**Figure 7:**
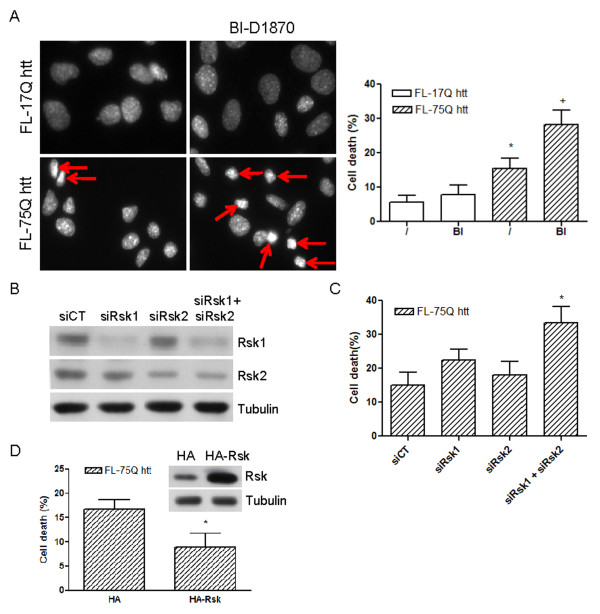
**Rsk protects against mhtt-induced cell death in striatal cells**. (A) STHdh^Q7/Q7 ^cells were transfected with full-length wild-type (FL-17Q htt) or mutant (FL-75Q htt) htt and then treated with the Rsk inhibitor BI-D1870 (0.1 μM; BI). Cell death was determined 72 hours after transfection using Hoechst 33258 staining and analyzing the condensation of the nucleus in transfected cells (cells expressing higher levels of htt). Representative photomicrographs show apoptotic nuclei in each condition. (B) STHdh^Q7/Q7 ^cells were transfected with siCT, siRsk1, siRsk2 or siRsk1+siRsk2. Cells were lysed 72 hours after transfection and the levels of Rsk1, Rsk2 and tubulin were analyzed by western blot. (C) STHdh^Q7/Q7 ^cells were co-transfected with FL-75Q htt and siCT, siRsk1, siRsk2 or siRsk1+siRsk2. Cell death was determined 72 hours after transfection using Hoechst 33258 staining. (D) STHdh^Q7/Q7 ^cells were co-transfected with FL-75Q htt and HA or HA-Rsk to study cell death 72 hours later using Hoechst 33258 staining. *Inset*, A representative western blot showing the overexpression of Rsk 72 hours after transfection of STHdh^Q7/Q7 ^cells with HA-Rsk. In all cases, results are shown as percentage of apoptotic nuclei *versus *total nuclei and are the mean ± SEM of three independent experiments performed in duplicate. Data were analyzed using two-way ANOVA followed by Bonferroni's *post hoc *test (A and C) and using a non-parametric *t*-test. In (A) *p < 0.05 as compared with FL-17Q htt-transfected cells and^+^p < 0.05 as compared with FL-75Q htt-transfected cells without BI-D1870 treatment; in (C) *p < 0.05 as compared with cells transfected with FL-75Q htt and siCT and in (D) *p < 0.05 as compared with cells transfected with FL-75Q htt and HA.

To evaluate whether elevated Rsk activity could exert a protective effect against mhtt-induced cell death, we study its protective capacity against mhtt-induced toxicity using pharmacological inhibition, knock-down and overexpression approaches. The expression of endogenous mhtt in immortalized STHdh^Q111/Q111 ^cells does not produce cell death. Thus, to induce mhtt toxicity, STHdh^Q7/Q7 ^cells were transfected with wild-type (FL-17Q htt) or mutant (FL-75Q htt) htt and cell death was assessed by Hoechst 33258 staining 72 hours after transfection. Overexpression of FL-75Q htt induced 16 ± 3% apoptotic cell death *versus *6 ± 2% apoptotic cell death observed in FL-17Q htt-transfected cells (Figure 7A). In parallel experiments we treated transfected cells with the Rsk inhibitor BI-D1870 (0.1 μM). The inhibition of Rsk exacerbated the toxic effect of FL-75Q htt expression and increased apoptotic cell death to 28 ± 4% (Figure 7A). In contrast, addition of BI-D1870 to FL-17Q htt-transfected cells did not alter cell death (Figure 7A). Our next goal was to know whether the protective role of Rsk was mediated by Rsk1, Rsk2, or by both isoforms. To address this issue, STHdh^Q7/Q7 ^cells were co-transfected with FL-75Q htt and with siRNAs against Rsk1 (siRsk1), Rsk2 (siRsk2) or both (siRsk1 + siRsk2). First, we checked that transfection with siRsk1 or siRsk2 separately decreased the protein levels of each isoform, and that the co-transfection with siRsk1 and siRsk2 reduced the expression of both isoforms (Figure 7B). The analysis of cell death showed that inhibition of Rsk1 or Rsk2 separately was not enough to increase the toxic effect of mhtt (Figure 7C). In contrast, the knock-down of both isoforms enhanced FL-75Q htt-mediated cell death (Figure 7C), similar to that observed after treatment with BI-D1870 (Figure 7A). To confirm the beneficial effect of Rsk in cells expressing mhtt, we overexpressed Rsk in cells transfected with FL-75Q htt by the co-transfection with HA-Rsk. The analysis of cell death 72 hours later revealed that Rsk overexpression reduced two-fold the cell death induced by mhtt (Figure 7D). Thus, we conclude that Rsk activity exerts a protective effect against mhtt-induced toxicity, and that both Rsk1 and Rsk2 isoforms are involved in this protective effect.

## Discussion

In this work, we provide for the first time evidence for a role of Rsk in HD. Within Rsk family, we analyzed Rsk1 and Rsk2, two isoforms that are broadly expressed in the brain, including the striatum, and whose expression levels are higher respect to other Rsk isoforms [1]. We observed increased protein levels of both Rsk1 and Rsk2 in the striatum of Hdh^Q111/Q111 ^and R6/1 mice, and in STHdh^Q111/Q111 ^cells, which are not dependent on mhtt protein levels as these HD models express different levels of mhtt. Thus, increased Rsk1 and Rsk2 protein levels is the result of the presence of mhtt, and we confirmed this hypothesis by showing that transfection of FL-75Q htt in STHdh^Q7/Q7 ^cells, M213 cells or striatal primary cultures elevated the levels of both Rsk isoforms. In addition, increased Rsk1-2 protein levels correlated with higher basal Rsk activity in STHdh^Q111/Q111 ^cells than in cells expressing wild-type htt. Interestingly, the inhibition of Rsk activity enhanced striatal cell death induced by transfection of mhtt (FL-75Q htt) in STHdh^Q7/Q7 ^cells. Moreover, we show that the overexpression of Rsk reduces considerably cell death in STHdh^Q7/Q7 ^cells transfected with FL-75Q htt. Altogether, these results indicate that elevated Rsk1-2 activity is an efficient mechanism to protect cells against mhtt toxicity. Thus, increased activity of Rsk1-2 could be a compensatory mechanism occurring in HD striatum.

Compensatory responses activated at early phases of HD are considered interesting targets to design neuroprotective therapies to inhibit the progression of neurodegeneration. Here, we analyzed Hdh^Q111/Q111 ^mice at 6 and 10 months of age, and R6/1 at 8 and 12 weeks of age, when they do not show motor dysfunction, but express cellular and molecular markers of HD pathology [22,24-28]. In addition, we studied the activity and the neuroprotective role of Rsk in STHdh cells, which derive from Hdh^Q111/Q111 ^embryos [29], and reproduce early events in the disease cascade [17]. We observed that increased Rsk activity is neuroprotective against mhtt-induced cell death since inhibition of its activity in FL-75Q htt-transfected STHdh^Q7/Q7 ^cells increased cell death. Moreover, we determined that this neuroprotective effect is due to an increase of Rsk activity in striatal cells expressing mhtt, since the inhibition of Rsk did not affect the viability of FL-17Q htt-transfected STHdh^Q7/Q7 ^cells. Confirming the protective role of Rsk against mhtt, we overexpressed Rsk in STHdh^Q7/Q7 ^cells and showed a protective effect against mhtt-mediated cell death. In addition, we show that both Rsk1 and Rsk2 are necessary to exert this protective role. Different studies in HD models have shown the regulation of other kinases as compensatory mechanisms activated in response of mhtt toxicity. These include Akt [17,30,31] and proteins closely related to Akt, such as the serum- and glucocorticoid-induced kinase [32]. Here, we show that in addition to these kinases, Rsk activity is also up-regulated in the presence of mhtt, and more importantly, that Rsk activity is neuroprotective against mhtt-induced cell death.

Present results also show that the increased activity of Rsk in striatal neurons expressing mhtt is mainly due to the action of PDK1, a kinase whose activity is independent of extracellular factors [3]. We detected increased phosphorylation of Rsk at Ser-221 (dependent on PDK1) and reduced phosphorylation of Rsk at Ser-380 (indirectly dependent on ERK1/2) in Hdh^Q111/Q111 ^mice striatum and STHdh^Q111/Q111 ^cells. In accordance with our results, STHdh^Q111/Q111 ^cells display elevated levels of phospho-PDK1 [17] and reduced levels of phospho-ERK1/2 [18] respect to STHdh^Q7/Q7 ^cells. In addition, using mutant forms of Rsk, we show that the activity of Rsk in STHdh^Q111/Q111 ^cells was considerably inhibited when PDK1-regulated, but not when ERK1/2-regulated residues, were mutated. Consistent with our observations, it has been suggested that PDK1 has the capacity to activate Rsk in an ERK1/2-independent manner [33]. Furthermore, and supporting the important role of PDK1-mediated phosphorylation to Rsk activity, PDK1 deficiency results in Rsk inactivation [34]. Although the neuroprotective role of Rsk has been classically associated with extracellular stimuli induced by trophic factors such as brain-derived neurotrophic factor [13] or epidermal growth factor [12] through the activation of ERK1/2, Rsk phosphorylation by PDK1 increases its activity to a higher extend than ERK1/2-dependent phosphorylation [3]. In HD, neurotrophic deprivation has been proposed as one of the mechanisms involved in the preferential loss of striatal neurons [28,35,36]. Thus, in the absence of trophic support, activation of Rsk through the basal activity of PDK1 could be a crucial mechanism to prevent cell death in HD.

The neuroprotective activity of Rsk is basically due to the wide range of proteins that it regulates. In the nucleus, Rsk phosphorylates and activates several transcription factors, some of them implicated in neuronal survival such as SRF [9], CREB [37] or NFκB [10,11]. Studies in non-neural cell lines showed that in the cytosol Rsk phosphorylates and inactivates pro-apoptotic proteins such as Bad [5], GSK-3β [6] or DAPK [7]. In this way, our data indicate that Rsk1-2 activity is elevated in the cytosol and in the nucleus of STHdh^Q111/Q111 ^cells. In the cytosol, increased Rsk activity correlated with an enhancement of phosphorylated Bad, whereas in the nucleus we observed increased levels of phospho-SRF. Changes in phospho-Bad and phospho-SRF were due, at least in part, to Rsk activation, since inhibition of Rsk significantly reduced the phosphorylation levels of both proteins in STHdh^Q111/Q111 ^cells. In STHdh^Q7/Q7 ^cells, the inhibition of Rsk produced a slight effect on SRF phosphorylation levels, and we did not detect an effect on Bad phosphorylation. Probably, this lack of effect on Bad phosphorylation is due the predominant nuclear activity of Rsk in unstimulated cells [1,2]. Overall, we propose that the neuroprotective effect of Rsk observed in the models studied here could be mediated by the inactivation of pro-apoptotic factors in addition to the activation of transcription factors that regulate the expression of anti-apoptotic proteins.

## Conclusions

In conclusion, here we provide evidences that the increase of Rsk1-2 levels is an early event taking place in striatal cells expressing full-length mhtt. Increased Rsk1-2 levels contribute to enhance Rsk activity. Interestingly, our results strongly support that increased Rsk activity in the presence of mhtt is mainly regulated by the basal activity of PDK1 and not by ERK1/2. Moreover, we show that the increase of Rsk1-2 activity observed in cells expressing mhtt could contribute to prevent mhtt-induced cell death. This is the first work showing a role for Rsk in HD, and we propose that therapies targeted to maintain Rsk activity would be a good approach for neuroprotection in HD.

## Methods

### HD mouse models

Homozygous mutant HdhQ^111/Q111 ^and wild-type Hdh^Q7/Q7 ^knock-in mice were obtained from mating between male and female Hdh^Q111/Q7 ^heterozygotes as described previously [23]. We also used R6/1 mice (B6CBA background) expressing the exon-1 of mhtt with 145 CAG repeats [38]. Mouse genotype was determined as described elsewhere [22]. CAG repeat length was determined by PCR amplification of the repeat using HD1 and HD2 fluorescently labeled primers as previously describe by the Huntington's Disease Collaborative Research Group [15], and subsequent size determination in an ABI 3100 analyzer. These results were double checked by Laragen, Inc. (Los Angeles, CA). All mice used in the present study were housed together in numerical birth order in groups of mixed genotypes, and data were recorded for analysis by microchip mouse number. Experiments were conducted in a blind-coded manner respect to genotype. Mice were genotyped by polymerase chain reaction as described previously [23]. The animals were housed with access to food and water *ad libitum *in a colony room kept at 19-22°C and 40-60% humidity, under a 12:12 hours light/dark cycle. All procedures were performed in compliance with the National Institutes of Health Guide for the Care and Use of Laboratory Animals, and approved by the Local Animal Care Committee of *Universitat de Barcelona *(99/01), and *Generalitat de Catalunya *(99/1094), in accordance with the Directive 86/609/EU of the European Commission.

### Cell cultures and pharmacological treatments

Conditionally immortalized striatal neuronal progenitor cell lines expressing endogenous levels of wild-type (STHdh^Q7/Q7^) or mutant (STHdh^Q111/Q111^) full-length htt with 7 and 111 glutamines, respectively, have been described elsewhere [26]. Cells were grown at 33°C in Dulbecco's modified Eagle's medium (DMEM; Sigma Chemical Co, St. Louis, MO) supplemented with 10% fetal bovine serum, 1% non-essential amino acids, 2 mM L-glutamine, and 400 μg/ml G418 (Geneticin; Invitrogen, Carlsbad, CA). M213 cells (conditionally immortalized striatal-derived neural stem cells) were grown as previously described [39]. Primary striatal cultures were obtained from 18-day old C57BL6 mouse embryos (Charles River, France). Striata were dissected as previously described [40,41]. Cells (50,000 cells/cm^2^) were seeded on plates pre-coated with 0.1 mg/mL poly-D-lysine (Sigma Chemical Co.) and cultured in Neurobasal medium supplemented with B27 (Gibco, Paisley, Scotland, UK) and glutamax at 37°C in a humidified atmosphere containing 5% CO_2_.

To measure Rsk activity and phosphorylated levels of Bad and SRF, the Rsk inhibitor BI-D1870 (0.1 μM, Boehringer Ingelheim Pharma GmbH & Co) was added during 4 hours to STHdh^Q7/Q7 ^and STHdh^Q111/Q111 ^cell cultures. For quantification of apoptosis, STHdh^Q7/Q7 ^and STHdh^Q111/Q111 ^cells were treated with BI-D1870 (0.1 μM) for 72 hours.

### DNA constructs and transfection

Full-length wild-type (FL-17Q htt) and mutant (FL-75Q htt) htt constructs were a gift from Drs. Fréderic Saudou and Sandrine Humbert (Institut Curie, Orsay, France). HA-Rsk, HA-Rsk T574A and HA-Rsk S381E were kindly provided by Dr. Dario Alessi (MRC Protein Phosphorylation Unit, Dundee, Scotland, UK). All DNA constructs were transfected using Lipofectamine 2000 (Invitrogen) as instructed by the manufacturer. Both STHdh^Q7/Q7 ^and M213 cells were transfected at 50% confluence, whereas primary striatal cultures were transfected at 5 days *in vitro*.

STHdh^Q7/Q7 ^cells were transfected using 7.5 pmol Rsk1 siRNA (Silencer^® ^Pre-designed siRNA, s73163 and s73164, Ambion, Applied Biosystems, Foster City, CA) and/or Rsk2 siRNA (Silencer^® ^Pre-designed siRNA, s99855 and s99857, Ambion) with Lipofectamine 2000 as instructed by the manufacturer and incubated during 4 hours. A non-targeting control siRNA (7.5 pmol) (Silencer^® ^Negative Control 1 siRNA, Ambion) was used to assess non-specific gene silencing effects. Cells were lysed or fixed 72 hours after transfection.

### Protein extraction and subcellular fractionation

STHdh^Q7/Q7 ^and STHdh^Q111/Q111 ^cells, with or without BI-D1870 treatment or Rsk knock-down, were washed once with phosphate-buffered saline (PBS), and total cellular proteins were extracted by incubating cells in lysis buffer containing 1% Triton X-100, 50 mM Tris-HCl (pH 7.5), 10 mM EGTA, 150 mM NaCl, protease inhibitors (2 mM phenylmethylsulfonyl fluoride (PMSF), 10 μg/μL aprotinin, 1 μg/μL leupeptin) and phosphatase inhibitor sodium orthovanadate (2 mM). Hdh^Q7/Q7 ^and Hdh^Q111/Q111 ^mice were deeply anesthetized and killed by decapitation at the age of 6 and 10 months and wt and R6/1 mice at 8 and 12 weeks of age. The brain was quickly removed and the striatum was dissected out and homogenized in lysis buffer (as above). All samples were centrifuged at 16 100 × *g *for 20 minutes at 4°C, the supernatants were collected and protein concentration was measured using the *Dc *protein assay kit (Bio-Rad Laboratories, Hercules, CA).

For subcellular fractionation, STHdh^Q7/Q7 ^and STHdh^Q111/Q111 ^cells were rinsed once with PBS and centrifuged at 800 *g *for 5 minutes. Pellets were homogenized in lysis buffer (10 mM Tris-HCl pH 7.5, 0.25 M sucrose, 2 mM PMSF, 10 μg/μL aprotinin, 1 μg/μL leupeptin, 2 mM sodium orthovanadate) and centrifuged at 3000 × *g *for 10 minutes. The resulting supernatant was centrifuged at 10 000 × *g *for 15 minutes to obtain a cytosol/light membrane supernatant that was further centrifuged at 100 000 × *g *for 15 minutes to obtain the cytosolic fraction (supernatant). The pellet resulting from the initial centrifugation was resuspended in lysis buffer and centrifuged at 800 × *g *for 10 minutes. The pellet, containing washed nuclear fraction, was then resuspended in lysis buffer (50 mM Tris-HCl pH 7.5, 150 mM NaCl, 10% glycerol, 1% Triton X-100, 10 mM EGTA, 2 mM PMSF, 10 μg/μL aprotinin, 1 μg/μL leupeptin, 2 mM sodium orthovanadate) and incubated for 30 minutes at 4°C in a tube rotator. Finally, after centrifuging for 15 minutes at 16 100 × *g*, the supernatant was collected and stored. Protein concentrations were determined as above.

### Western blot

Western blot was performed as described elsewhere [41]. The following primary antibodies were used: anti-Rsk1, anti-Rsk2, anti-phospho-Rsk (Ser-221) and anti-phospho-Rsk (Ser-380) (all 1:500; Santa Cruz Biotechnology, Santa Cruz, CA), anti-htt monoclonal 2166 (1:1000; Millipore Bioscience Research reagents, Temecula, CA), anti-phospho-SRF (Ser-103), anti-SRF, anti-phospho-Bad (Ser-112) and anti-Bad (all 1:1000; Cell Signaling Technology, Beverly, MA), and anti-HA (1:1000; Sigma-Aldrich, Saint Louis, MO). Loading control was performed by reprobing the membranes with anti-NeuN (1:1000; Chemicon, Temecula, CA), anti-α-tubulin (1:100.000; Sigma-Aldrich) or anti-actin (1:10 000; MP Biochemicals, Aurora, OH).

### Immunocytochemical staining, confocal microscopy analysis, and Rsk localization

Cells were fixed in 4% paraformaldehyde for 10 minutes, incubated with 0.2 M glycine for 20 minutes and permeabilized in 0.1% saponin for 10 minutes. Blocking was performed with 1% BSA in PBS for 1 hour. Specimens were incubated overnight with the primary antibodies (all 1:100): anti-htt monoclonal 2166, anti-Rsk1 and anti-Rsk2. Afterwards, specimens were incubated with subtype-specific fluorescent secondary antibodies: Cyanine 3 anti-rabbit (1:200; Invitrogen), rhodamine-conjugated anti-mouse (1:200; Jackson ImmunoResearch, West Grove, PA) and Alexa 647 anti-mouse (1:150; Invitrogen).

For quantification of Rsk in htt-transfected cells, immunocytochemistry was performed 24 (striatal primary cultures) or 48 (striatal knock-in and M213 cells) hours after transfection. Quantification of Rsk1 and Rsk2 was performed by confocal microscopy (Leica, Mannheim, Germany) as previously described [42]. Values were expressed as a ratio between the sums of Rsk1 or Rsk2 positive pixels *versus *cell area. For each condition, 30-40 cells were randomly selected. To study the localization of Rsk1 and Rsk2, STHdh^Q7/Q7 ^and STHdh^Q111/Q111 ^cells were fixed at 80% confluence and processed for immunocytochemistry against Rsk1 or Rsk2. At least 250 cells were evaluated for each condition.

### Rsk activity assay

To measure Rsk activity, the assay was performed in STHdh wild-type or mutant cells at 80% confluence or 24 hours after transfection with Rsk constructs, as described previously [23]. Briefly, immunoprecipitation of Rsk was performed by incubation of total protein extracts (100 μg) with anti-Rsk1 and anti-Rsk2 antibodies, 1 μg each. Then, immunoprecipitates were incubated for 15 minutes at 30°C under continuous agitation with the assay mixture buffer containing the substrate peptide and the mixture of ATP and [γ-^32^P] ATP (PerkinElmer, Boston, MA). Reactions were terminated and analyzed as described elsewhere [43]. Incubation with BI-D1870 was used to assess the specificity of Rsk activity assay.

### Quantification of apoptosis

STHdh^Q7/Q7 ^cells transfected with FL-17Q htt or FL-75Q htt, with or without Rsk siRNAs, or Rsk DNA plasmid transfection or BI-D1870 treatment were processed for immunocytochemistry against htt as described above. Finally, cells were washed twice in PBS and stained with Hoechst 33258 (1 μg/mL; Molecular Probes, Inc, Eugene, OR) for 5 minutes. After washing twice with PBS the coverslips were mounted with mowiol. Nuclear DNA staining was observed with a fluorescence microscope (Olympus). Transfected cells were detected by the overexpression of htt respect to non-transfected cells. Condensed or fragmented nuclei were counted as apoptotic. At least 100 cells were evaluated for each condition in each independent experiment.

### Statistical analysis

Statistical analysis was performed by using the one- or two-way analysis of variance (ANOVA) followed by Bonferroni's post-hoc test, or the unpaired Student's *t*-test, as appropriate and indicated in the figure legends. The analysis of Rsk distribution by immunocytochemical staining was performed using the chi square. A value of p < 0.05 was accepted as denoting statistical significance.

## Abbreviations

The abbreviations used are: CREB: cAMP response element binding protein; CTKD: C-terminal kinase domain; DAPK: death-associated protein kinase; ERK: extracellular signal-regulated kinase; GSK: glycogen synthase kinase; HD: Huntington's disease; htt: huntingtin; Hdh^Q7Q/7^: wild-type huntingtin mouse; Hdh^Q111/Q111^: mutant huntingtin mouse; mhtt: mutant huntingtin; NTKD: N-terminal kinase domain; PDK1: 3-phosphoinositide-dependent protein kinase-1; Rsk: 90-kDa ribosomal S6 kinase; SRF: serum response factor; STHdh^Q7/Q7^: striatal wild-type huntingtin cells; STHdh^Q111/Q111^: striatal mutant huntingtin cells; WT: wild-type.

## Competing interests

The authors declare that they have no competing interests.

## Authors' contributions

XX, and JA conceptualized the study. XX, EPN and JA wrote the manuscript. XX, MAH, LR and AS carried out experiments. All authors read and approved the final manuscript.
